# Adolescents’ attitudes towards healthy eating: A scale development study

**DOI:** 10.1371/journal.pone.0334945

**Published:** 2025-10-27

**Authors:** Remzi Eşkil, Kezban Gülşen Eşkil, İmdat Yarım

**Affiliations:** Faculty of Sport Sciences, Gazi University, Turkey; King Khalid University, EGYPT

## Abstract

The aim of this study is to develop a measurement tool that can reliably and validly measure adolescent individuals’ attitudes toward healthy eating. The study group consisted of 1,006 individuals, including 495 males and 511 females, aged between 11 and 17 years. In this study, an exploratory sequential design was applied. A semi-structured interview form was used to create the item pool, and compositions were written. The “Davis Technique” was employed to assess the content validity of the items. For data analysis, SPSS 25.0 was used for EFA and reliability analysis, and Lisrel 8.7 was utilized for CFA. Based on the result of EFA, a structure consisting of 4 factors and 18 items was formed. The total variance explained is 59.05. According to CFA analysis, factor loadings range from .43 to .81, and X2/df = 1.65, RMSEA = .040 were found. Furthermore, the NFI, NNFI, PNFI, CFI, IFI, GFI, AGFI, PGFI, and RFI fit indices were found to be excellent and within the good range. In the analysis of the lower and upper groups of the scale (27%), statistically significant differences were observed in all items (p < .01). To test the reliability of the scale, Cronbach’s Alpha, Spearman-Brown (Split-half), and Guttman Lambda-6 coefficients were examined. The scale sub-dimension correlations ranged from .526 to .129. In conclusion, the developed scale demonstrates that it can validly and reliably measure adolescents’ attitudes toward healthy eating.

## Introduction

Growth and development is a dynamic process. Many environmental factors influence this period, one of which is nutrition [1]. During this period, nutrition must be adequate and balanced in order for the individual to continue to grow and develop [2], and nutrients must be consumed at the right times and in the right way [3]. Healthy nutrition is a diet that minimizes processed foods, frozen fats, and added sugars, and includes whole grain products, fruits, vegetables, lean protein products, and adequate fluid intake [4]. Adequate nutrient intake and good eating habits are very important for optimal development during adolescence [5]. During this period, rapid changes and growth occur in the human body [6], and individuals experience the fastest social, emotional, physical, and sexual development [7]. The World Health Organization defines adolescence as the period between the ages of 10 and 19 and considers it a critical time for laying the foundations for a healthy life [8]. Individuals of this age begin to make their own decisions [9]. These decisions can lead to changes in the eating habits of adolescents. Additionally, adolescents’ healthy eating habits can be influenced by various factors such as culture, socioeconomic status, family attitudes, and peer influence. A family with a low socioeconomic status often has limited access to fresh food and may be forced to resort to processed foods that are high in calories but low in nutritional value [10]. The family’s attitude toward nutrition can play an important role in shaping eating and drinking habits within a given culture. The culture in which individuals live can either encourage or discourage them from eating a balanced diet [11]. Whether an individual has a positive or negative attitude toward healthy eating is very important in this regard. The concept of attitude is defined as an individual’s affective, behavioral, and mental response to any object or themselves [12]. Attitudes are an individual’s negative or positive self-assessment of a particular behavior. As a result of this perceived norm, the individual may or may not engage in the behavior [13]. From this perspective, it is important for individuals to develop positive attitudes toward adequate and balanced nutrition from an early age. Morever, a sedentary lifestyle, excessive screen time, lack of physical activity, and poor nutrition can lead to excessive weight gain in adolescents [4]. Adolescent obesity has been linked to cardiovascular disease, diabetes, and similar diseases in adulthood [14]. According to data from the World Health Organization, 37 million children under the age of 5 and 390 million children and adolescents between the ages of 5 and 19 were overweight in 2022. The obesity rate in the 5–19 age group has risen from 2% to 8%, and being overweight is now a major problem not only in high-income countries but also in low- and middle-income countries [15]. Such negative situations can be overcome by adolescents’ attitudes and awareness of adequate and balanced nutrition [16]. Healthy eating habits formed during adolescence can reduce the risk of diseases such as diabetes, cardiovascular diseases, and obesity, and contribute to individuals’ lifelong health [17]. These attitudes are very important for reducing future health risks, reducing the increasing obesity among children and adolescents around the world, and ensuring the health of future generations. A review of the literature reveals the “infant feeding attitude scale” [18], the “nutrition and behavior attitude scale for bariatric surgery patients” [19], and a nutrition attitude scale for adults [20], but no attitude scale for adolescents regarding healthy eating has been found. It is believed that this aspect of our study will fill a significant gap in the field. Based on these findings, and taking into account the developmental characteristics specific to adolescence, the aim was to develop a valid and reliable measurement tool to assess adolescents’ attitudes toward healthy eating, which is closely related to healthy lifestyle habits and forms the basis for long-term health behaviors.

## Materials and methods

### Research model

**Fig 1:**
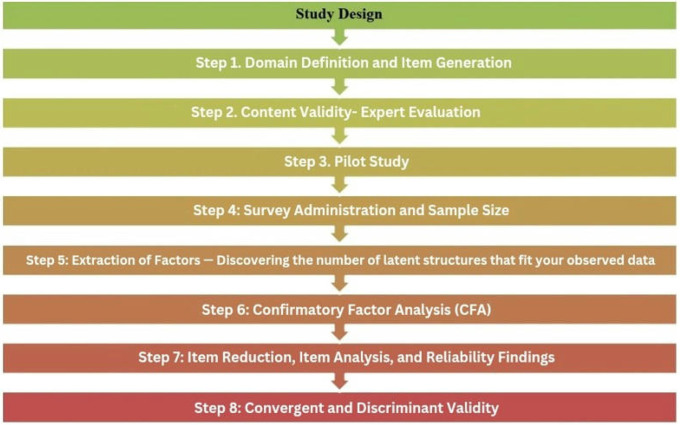
Study design.

In this study, a mixed research design was used. Mixed methods research integrates both quantitative and qualitative data, where the data sources complement, support, and validate each other. In the exploratory sequential design, the researcher begins with qualitative data collection and then proceeds to quantitative data. This design is most commonly used to explore phenomena, design a scale tool, and subsequently test the developed tool [21] (Fig 1).

#### Phase 1. Item generation.

**Step 1. Domain definition and item generation**

In scale development research, several essential steps must be followed: clearly defining the structure to be measured, generating an item pool, determining the method of measurement, having the draft item pool reviewed by experts, conducting a pilot study, administering the items to the scale development sample, and refining the scale length to an appropriate form. In the scale development process, it is first necessary to identify the target population and thoroughly examine the theoretical structure to be measured. This forms the foundation for developing a valid and reliable measurement instrument [22]. Attitudes influence our tendency to accept or reject objects, ideas, and groups, revealing our feelings toward or against these objects, ideas, and groups and affecting the outcome of our behavior [23]. Attitudes have three dimensions: cognitive, affective, and behavioral, and there is a dynamic relationship between these dimensions [24]. When creating the scale item pool, it was made sure that the items covered the cognitive, affective, and behavioral dimensions and that positive and negative statements were balanced. Since items that did not have attitude characteristics, had language and meaning issues, or contained vague or ambiguous statements were to be eliminated, the item pool was made as comprehensive as possible. After defining the domain, there are two ways to create items: deduction and induction. The induction method can be performed by reviewing the literature and evaluating existing scales. This method is also known as grouping and classification. In the induction method, on the other hand, qualitative data obtained through methodologies such as focus groups and individual interviews can be used [25]. In this study, the inductive method was used. A semi-structured interview form was developed by experts in the field to create an attitude scale for adolescents regarding healthy eating. In this form, questions related to adequate and balanced nutrition were asked to 29 volunteers. Additionally, 34 volunteers were asked to write compositions related to adequate and balanced nutrition. This semi-structured interview form, written compositions, and nutrition attitude scales developed in the literature were examined, and taking into account the concept and structure of attitude, a draft item pool consisting of a total of 72 items was created.

**Step 2. Content Validity- Expert Evaluation**

Expert opinion was sought regarding the validity of the content. The purpose of the expert opinion was to ensure the content validity of the items on the scale and to remove inappropriate items [26]. The 72-item draft scale was created, and expert opinion was sought by writing “1- Inappropriate, 2- The item needs to be seriously reviewed, 3- Appropriate, but minor corrections may be necessary, 4- Completely appropriate” next to each item. It was then evaluated by a total of five experts: a language specialist in the field of Turkish Language and Literature who assessed its linguistic appropriateness, three specialists in the field of nutrition and dietetics, and an assessment specialist from the guidance and psychological counseling department. The experts made corrections in terms of grammatical structure, clarity of meaning, and conceptual accuracy. The content validity of the items was evaluated using the “Davis Technique.” The internal consistency validity index (I-CVI) was set at a minimum of .80, and the 8 items that did not reach this value were removed from the scale [27]. As a result of these procedures, the draft scale consisting of 64 items took its final form. The scale items were rated on a 5-point Likert scale ranging from “Strongly Disagree (1)” to “Strongly Agree (5)”.

#### Phase 2. Scale development.

**Step 3. Pilot Study.**

The pilot study is conducted face-to-face with a group of 15–20 people to test the comprehensibility of the scale items, and its purpose is to remove items that are not understood from the scale [28]. This study was conducted on 34 people aged 12–15. Four items that were not understood in the draft scale were removed from the scale, and 60 items were made ready for application.

### Data collection

Gazi University Ethics Committee (2024−1315) ethical committee approval and necessary permissions were obtained. Signed consent forms were obtained from both parents and participants. All participants were informed in advance about the purpose and summary of the study, the requirements for participation, the procedure, the voluntary nature of participation, the expected risks and benefits for individuals. In particular, the confidentiality of participants was ensured. Participation was entirely voluntary, and participants were informed that they could withdraw from the study at any time. Additionally, the study was conducted in accordance with the Helsinki Declaration. The study topic was explained to the students in an understandable manner, and they were then asked to complete the scale form. The data collection step of the study was completed between April 17 and May 17, 2025.

**Step 4: Survey Administration and Sample Size**

The study group consisted of a total of 1,006 individuals, including 495 males and 511 females aged between 11 and 17 years. A criterion sampling method was used in the study. In this method, individuals and situations that meet predefined criteria can be selected and determined by the researcher [29]. When determining the research group, students aged 11–17 who volunteered to participate were included in the process; students who did not meet these conditions were excluded. When reviewing the literature, it is recommended that the sample size be 5, 10, or 20 times the number of items [26,30,31]. Additionally, it is stated that a sample size of 1,000 individuals is ideal, 500 individuals is very good, 200 individuals is sufficient, and 50 individuals is very weak [32]. Since the research group consisted of a total of 1,006 individuals, it was found to be suitable for both situations. In the literature, it is stated that when an adequate sample size is achieved, this sample can be randomly divided into two subgroups [22]. In this study, participants were randomly divided into two groups: Group 1 underwent exploratory factor analysis (EFA), and Group 2 underwent confirmatory factor analysis (CFA).

#### Research Group 1.

This group of the study consisted of a total of 606 participants, including 296 males and 310 females aged between 11 and 17 years. Descriptive factor analysis (CFA), Cronbach’s alpha reliability coefficient calculation, and item analyses were performed using the data of the participants in this group.

#### Research Group 2.

This group of the study consisted of a total of 400 people, including 199 males and 201 females between the ages of 11 and 17. Confirmatory Factor Analysis (CFA), Cronbach’s alpha reliability coefficient calculation, and item analyses were also performed on the data of this group.

## Results

### Data analysis

Forms that were filled out incorrectly or incompletely were not included in the analysis. The analysis continued with data collected from a total of 1,006 students in the final scale. Exploratory Factor Analysis (EFA) and Confirmatory Factor Analysis (CFA) were performed to provide evidence of the validity of the scale developed to measure adolescents’ attitudes toward healthy eating. SPSS 25.0 software was used for CFA and reliability analysis. In addition, at this stage, the Kaiser Meyer Olkin (KMO) and Bartlett Sphericity tests were employed to determine the suitability of the data for factor analysis. The Lisrel 8.7 program was used for CFA. In this step, the chi-square, df, RMSEA values, and NFI, NNFI, PNFI, CFI, IFI, GFI, AGFI, PGFI, and RFI Fit Index values were examined from the test scores obtained.

Step 5: Extraction of Factors—Discovering the number of latent structures that fit your observed data

### Exploratory Factor Analysis (EFA)

Exploratory Factor Analysis (EFA) is a common statistical technique that reduces a large number of interrelated variables into a smaller number of meaningful and independent factors [33]. It allows items measuring the same trait to be grouped under relevant factors [22] and enables the removal of items in the measurement tool that do not adequately represent the structure or load on more than one factor [34].

The study utilized the KMO-Kaiser-Meyer-Olkin Sample Adequacy Test and Bartlett’s Test of Sphericity.

According to **Table 1**, the Kaiser-Meyer-Olkin (KMO) measure of sampling adequacy was .900, and the Bartlett’s Test of Sphericity chi-square value was measured as χ² = 13595.292 (df = 1770, p < .00).

**Table 1:** Principal Component Analysis (PCA) Results.

KMO Sample Adequacy Value		.900
Bartlett’s Test of Sphericity	Approximate Chi-Square	13595.292
	Degrees of Freedom (df)	1770
	Significance Level (p)	.000

**Fig 2:**
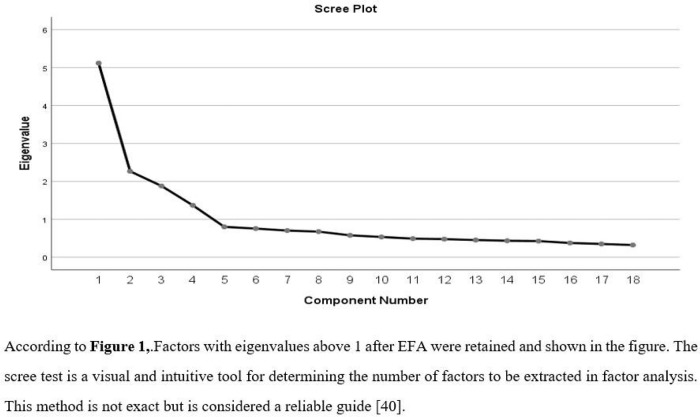
Scree plot graph.

Before proceeding to factor analysis, the KMO (Kaiser-Meyer-Olkin) coefficient and Bartlett’s Sphericity Test were applied to evaluate the suitability of the data. A review of the literature indicates that a KMO value above .50 is indicative of data suitability for factor analysis [35]. Bartlett’s Sphericity Test is a test that examines whether the data is normally distributed. A significant result of this test (p < .05) demonstrates that the data is normally distributed. As a result of these analyses, with a KMO value of .90 and a significant Bartlett’s Sphericity Test (p < .05), it can be said that the scale data is suitable for factor analysis. Furthermore, a KMO value above .90 is considered an excellent result [36]. In factor analysis, the Principal Components Analysis (PCA) technique was used. This method is a common starting technique in factor analysis, used to reduce a large number of variables to a smaller number of components. Additionally, the Promax rotation method was used.The criteria used in the Exploratory Factor Analysis (EFA) are as follows: the Kaiser criterion was adopted, retaining only factors with eigenvalues of at least 1 [35]. A factor loading cutoff value of .40 was applied, as recommended by [37,38]. Items that did not load above .40 on any factor, or that loaded above .40 on multiple factors with less than a .20 difference between the loadings, were excluded from the scale. Based on these criteria, a total of 42 items (Items 1, 2, 3, 4, 6, 8, 10, 11, 13, 14, 15, 16, 18, 20, 21, 22, 27, 28, 29, 31, 32, 33, 35, 36, 37, 38, 39, 40, 41, 42, 43, 44, 45, 46, 47, 48, 50, 51, 57, 58, 59, and 60) were removed due to ambiguous or low factor loadings (Fig 2).

According to **Table 2**, it is seen that the Healthy Eating Attitude Scale for adolescents consists of four factors and 18 items.

**Table 2:** Exploratory Factor Analysis (EFA) Results of the Attitude Scale.

Final Item No (EFA)	Items	Factor1	Factor 2	Factor3	Factor 4	Communalities (Shared Factor Variance)
I9	Balanced and adequate nutrition affects me positively.	.890				.655
I7	Balanced and adequate nutrition is important for everyone.	.748				.558
I24	Balanced nutrition makes me feel energetic.	.703				.618
I25	Nutrients are important for a healthy life.	.677				.534
I30	Balanced nutrition strengthens my immune system.	.610				.521
I55	I like eating fruits.		.787			.631
I26	I am happy when I eat fruit at every meal.		.783			.510
I12	Eating fruit at least 3–4 days a week is important to me.		.746			.600
I19	I like to eat fruit for balanced nutrition.		.654			.583
I56	I eat vegetables at least 3 days a week.		.538			.411
I53	I know foods containing carbohydrates.			.861		.728
I52	I know foods containing protein.			.837		.689
I54	I know foods containing vitamins and minerals.			.807		.686
I23	I know which vitamins are in which fruits.			.581		.410
^*^ *I34*	*I love to eat sugary foods.*				.839	.697
^*^ *I5*	*I feel happy when I eat junk food (chocolate, chips, biscuits, etc.).*				.786	.632
^*^ *I49*	*I like to eat sweets excessively.*				.762	.599
^*^ *I17*	*I like carbonated drinks (cola, energy drinks, etc.).*				.752	.568
	Eigenvalues	5.118	2.267	1.879	1.367	
	Variance %	28.43	12.59	10.43	7.59	
	Total Variance %	59.05				

* Items marked with * are reverse-coded items.

In the study, the factors were named using a content-based approach. The factors were selected in line with the theoretical foundations of the attitude concept [39], and the contents of the items in the factors were taken into account to ensure consistency [22,40]. 1. The first factor explains 28.432% of the variance and is named the “Affective Subdimension” based on the literature. 2. The second factor explains 12.594% of the variance and is named the “Positive Behavioral Subdimension.” 3. Factor explains 10.438% of the variance and is named “Cognitive Subdimension. 4. Factor explains 7.593% of the variance and is named “Negative Behavioral Subdimension. The behavioral sub-dimension emerged in two separate factors, labeled positive and negative, and these factors were supported by expert opinion in accordance with the principle of thematic similarity for content validity [41]. The total variance explained is 59.05%. It has been stated that a variance explained value between 40% and 60% is sufficient in the social sciences [24]. According to the EFA results in the study, the variance explained values are at an acceptable level. It has been noted that the minimum factor loading for each item should be at least .32 [42]. In the study, factor load values (cutoff value) were set at a minimum of .40.The factor load values of the items in the measurement tool range from .53 to .89. In addition, the common factor variance, which indicates how much of the variance each factor explains, must be greater than .03 [43]. Looking at the table above, it can be said that each item is greater than .04 and explains sufficient variance for a four-factor structure. Based on these findings, it can be shown as evidence that the items included in the scale strongly represent their dimension.

#### Phase 3. Scale evaluation.

**Step 6: Confirmatory Factor Analysis (CFA)**

**Fig 3:**
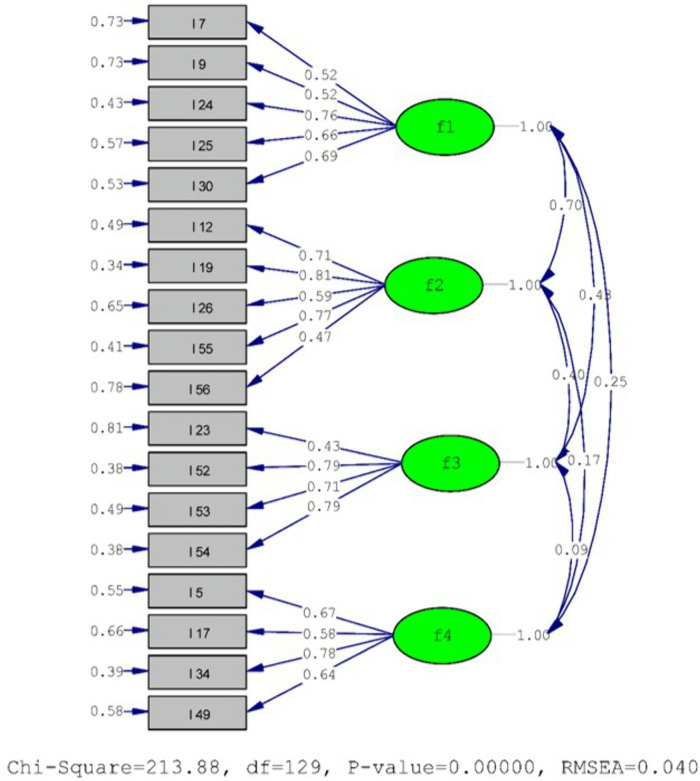
Confirmatory Factor Analysis (CFA) model of the healthy eating scale for adolescents.

After obtaining a structure consisting of 4 factors and 18 items as a result of exploratory factor analysis, CFA was performed using the Lisrel 8.7 program on a total of 400 student data, including 199 male and 201 female students, from the second study group to determine the degree of suitability of the obtained structure. CFA is used as a test method to validate the structure represented in exploratory factor analysis [44,45] (Fig 3).

According to **Table 3**, To establish the validity of the attitude scale toward healthy eating among adolescents, confirmatory factor analysis was performed on an 18-item, 4-factor scale. The fit indices were found to be excellent and within the acceptable range. A χ2/df value of less than 2 indicates excellent model-data fit [26].

**Table 3:** Criteria and Results of the Confirmatory Factor Analysis (CFA) Fit Indices.

Fit Index	Excellent Fit	Acceptable Fit	Nutrition Scale Result	Fit Level
^1^X2/ df	**0 < X2/ df < 2**	2 < X2/ df < 3	1.65	Excellent
^4^RMSEA	**.00 < RMSEA < .05**	.05 < RMSEA < .10	.04	Excellent
^3^NFI	**.95 < NFI < 1.00**	.90 < NFI < .95	.95	Excellent
^3^NNFI	**.95 < NNFI<1.00**	.90 < NNFI < .95	.97	Excellent
^2^PNFI	.95 < PNFI<1.00	**.50 < PNFI < .95**	.80	Good
^3^CFI	**.95 < CFI < 1.00**	.90 < CFI < .95	.98	Excellent
^2^IFI	**.95 < IFI < 1.00**	.90 < IFI < .95	.98	Excellent
^3^GFI	.95 < GFI < 1.00	**.85 < GFI < .95**	.94	Good
^2^AGFI	**.90 < AGFI<1.00**	.80 < AGFI < .90	.92	Excellent
^5^PGFI	.95 < PGFI<1.00	**.50 < PGFI < .95**	.71	Good
^3^RFI	.95 < RFI < 1.00	**.90 < RFI < .95**	.94	Good

^1^[26] ^2^[46–48] ^3^[46, 47, 49–52] ^4^[26,42,53,54] ^5^[55]

According to **Table 4**, a statistically significant difference was found between the lower and upper 27% groups in the total subdimension scores during the item analysis of the scale, which can be considered evidence of item discriminability [28]. Based on the CFA results of the scale, t-values were examined to determine the significance of each item. The findings indicate that the t-values ranged from 6.63 to 13.36. The t-values for all items were found to be statistically significant at the p < .01 level. These results demonstrate that the 18-item measurement tool provides good fit and is suitable for use following the CFA.

**Table 4:** T-Test Results for the Lower 27% and Upper 27% groups on the healthy eating attitude scale for adolescents.

Subdimension	Item No	Upper %27 Group *n*=	Lower %27 Group *n*=	t	p
Affective Subdimension		$\bar{x}$ss	$\bar{x}$ss		
	I7	4.72 ± .561	3.87 ± .958	7.97	.000^**^
	I9	4.73 ± .485	3.85 ± .915	8.82	.000^**^
	I24	4.75 ± .495	3.31 ± 1.056	12.78	.000^**^
	I25	4.74 ± .500	3.68 ± .783	11.90	.000^**^
	I30	4.78 ± .460	3.61 ± .936	11.62	.000^**^
Positive Behavioral Subdimension	I12	4.69 ± .571	3.42 ± 1.042	11.17	.000^**^
	I19	4.87 ± .412	3.49 ± .991	13.36	.000^**^
	I26	4.28 ± .915	2.89 ± 1.147	9.83	.000^**^
	I55	4.93 ± .263	3.71 ± 1.051	11.63	.000^**^
	I56	4.31 ± .932	2.70 ± 1.052	11.84	.000^**^
Cognitive Subdimension	I23	3.88 ± 1.039	2.56 ± 1.097	9.11	.000^**^
	I52	4.37 ± .827	3.18 ± 1.151	8.76	.000^**^
	I53	4.20 ± .873	3.05 ± 1.097	8.58	.000^**^
	I54	4.32 ± .783	3.12 ± 1.021	9.72	.000^**^
Negative Behavioral Subdimension	I5	2.69 ± 1.228	1.77 ± .992	6.03	.000^**^
	I17	3.54 ± 1.314	1.98 ± 1.094	9.45	.000^**^
	I34	3.19 ± 1.120	2.12 ± 1.030	7.27	.000^**^
	I49	3.26 ± 1.139	2.24 ± 1.118	6.63	.000^**^

** p < .01

**Step 7: Item Reduction, Item Analysis, and Reliability Findings**

In order to evaluate the reliability of the scale, Cronbach’s alpha coefficient, alpha values calculated when items were deleted, and item-total correlations were examined within the framework of Classical Test Theory (CTT) [56].

In **Table 5**, the internal consistency coefficient α for the subscales of the scale was found to be between .76 and .81, and α was .83 for the entire scale. This value indicates that the reliability level of the scale is high. The corrected item-total correlations for each item ranged from .42 to .66.

**Table 5:** Cronbach’s Alpha and item-total correlations.

Factors	Items	$\bar{x}$ss	Corrected Item-Total Correlation	Cronbach’s Alpha if Item Deleted
Affective Subdimension **α = .81**	I7	4.32 ± .863	.570	.783
	I9	4.30 ± .859	.596	.775
	I24	4.16 ± .885	.657	.755
	I25	4.24 ± .788	.587	.778
	I30	4.23 ± .837	.581	.779
Positive Behavioral Subdimension **α = .76**	I12	4.18 ± .922	.600	.695
	I19	4.27 ± .882	.575	.705
	I26	3.51 ± 1.144	.479	.741
	I55	4.34 ± .827	.637	.690
	I56	3.66 ± 1.183	.434	.762
Cognitive Subdimension **α = .78**	I23	3.28 ± 1.029	.420	.816
	I52	3.76 ± .999	.640	.706
	I53	3.67 ± .987	.664	.694
	I54	3.67 ± 1.003	.656	.698
Negative Behavioral Subdimension **α = .79**	I5	2.35 ± 1.143	.599	.740
	I17	2.56 ± 1.298	.564	.758
	I34	2.69 ± 1.147	.667	.707
	I49	2.70 ± 1.279	.577	.751

Total Scale **α =.83**

According to **Table 6**, in order to test the reliability of the adolescent individuals’ attitude scale towards healthy eating, Cronbach’s alpha coefficient, Spearman-Brown (Split-half) and Guttman Lambda-6 coefficients were examined. Reliability refers to the consistency of the items in a questionnaire or test and the extent to which the scale reflects the issue of interest. The alpha coefficient is a correlation-based model and gives the alpha number. This number, which can take values between 0 and 1, is equal to Kr-20. The split-half model divides the scale into two equal parts and examines the correlation between these parts. Guttman’s covariance or variance approach is used to calculate reliability, yielding a coefficient ranging from 1 to 6 [33]. Reliability coefficients vary between .73 and .88, and values above .70 are considered sufficient by researchers [26,33,56–58].

**Table 6:** Reliability Analysis Results of Adolescents’ Attitudes Toward Healthy Eating.

Scale Subdimensions	Number of Items	Cronbach Alpha (α)	Spearman-Brown Split-half	Gutman (λ₆)
Affective Subdimension	5	.81	.78	.78
Positive Behavioral Subdimension	5	.76	.73	.73
Cognitive Subdimension	4	.78	.80	.75
Negative Behavioral Subdimension	4	.79	.77	.74
Total Scale	18	.83	.88	.87

**Step 8: Convergent and Discriminant Validity**

According to **Table 7**, scale sub-dimension correlations were found to be positive between .526 and .129 (p < .01). Convergent validity was determined by analyzing the AVE and CR values of the four factors. AVE values ranged from .50 to .62, while CR values ranged from .83 to .87. Regarding discriminant validity, the √AVE values for all factors were found to be greater than their corresponding correlation coefficients.

**Table 7:** Correlation Values Between Factors, Convergent and Discriminant Validity.

Subdimensions	1	2	3	4
1 Affective Subdimension	1	.526^**^	.353^**^	.195^**^
2 Positive Behavioral Subdimension		1	.324^**^	.204^**^
3 Cognitive Subdimension			1	.129^**^
4 Negative Behavioral Subdimension				1
√ AVE	.73	.71	.78	.79
AVE	.54	.50	.61	.62
CR	.85	.83	.86	.87

**p < .01

## Discussion

The aim of this study is to develop a measurement tool that can reliably and validly measure adolescent individuals’ attitudes toward healthy eating. The 5-point Likert-type scale consists of four subdimensions—“Affective Subdimension,” “Positive Behavioral Subdimension,” “Cognitive Subdimension,” and “Negative Behavioral Subdimension”—and 18 items. During the scale development process, the target population was first identified, and the literature related to attitude characteristics, sub-dimensions, and theoretical structure was reviewed [24,28]. This iessential for developing a valid and reliable measurement tool [22]. When creating the item pool, semi-structured interview forms and compositions written by students were utilized. When creating the scale items, items were written for the cognitive, affective, and behavioral subdimensions of the attitude concept. The inductive method was used in this study [25]. A total of 72 items were written and expert opinion was obtained. Seeking expert opinion is crucial for content validity [26,59]. The content validity of the items was assessed using the “Davis Technique,” and the content validity index (I-CVI) was determined to be at least .80 [27]. The 8 items that fell below this value were removed from the scale. A pilot study was conducted on a group of 34 people. The aim here was to remove items that were not understood from the scale [28,60], and the final version of the 60-item draft scale was obtained after 4 items were removed from the scale. A criterion sample was used in the study; those who met the criteria were included in the study, and those who did not were excluded [29]. Furthermore, regarding the determination of the sample size, there are views in the literature suggesting that the sample size should be 5-10-20 times the number of items [26,30,31] or that the sample size should be between 50 and 1,000 individuals [32]. From this perspective, it can be said that the sample size of the study is adequate. Exploratory Factor Analysis (EFA) was performed to ensure the validity of the scale. A KMO value above .50 and a Bartlett’s Sphericity Test p < .05 are significant criteria. The KMO value was .90, which is proof that it is within the perfect range for factor analysis [36]. In the factor analysis, the PCA (Principal Component Analysis) method was employed along with the promax rotation technique. The promax rotation technique is a simple structure that clarifies which variables are related to each factor and which are not when the factors are interrelated [42]. The Kaiser criterion was used as a criterion when performing EFA in the study. The Eigen Value for each factor was taken as at least 1 [35]. Then, the number of factors was visually verified using the Scree test. This method is not definitive but is considered a reliable guide [61]. From this perspective, a four-factor structure was obtained from both angles. The cutoff value in factor analysis was set at .40. These values are also recommended by [37,38]. The factor loadings of the scale items range from .53 to .89. It has been stated that the minimum factor loading required for a scale item is .32 [42]. In this respect, it can be said that the factor loadings are sufficiently high and represent the sub-dimension well. The total variance explained by factor analysis is 59.05%. It has been noted that a variance explained value between 40% and 60% is sufficient in the social sciences [24]. Based on these results, the variance explained values are at an acceptable level. The EFA yielded a structure with 4 factors and 18 items. To test the suitability of the obtained structure, CFA was performed using the Lisrel 8.7 program. CFA is used to validate the structure represented in exploratory factor analysis [44,45]. A χ2/df value less than 2 and an RMSA value less than .050 can be used as evidence that the model fits the data almost perfectly [26]. In this study, a χ2/df value less than 2 and an RMSA value less than .050 can be used as evidence that the model fits the data almost perfectly. Confirmatory factor analysis was applied to examine the fit indices NFI, NNFI, PNFI, CFI, IFI, GFI, AGFI, PGFI, and RFI, and these values were found to be excellent and within the good range [26,42,46–55]. A statistically significant difference was observed in the total scores of the lower and upper groups (27% each) in the item analysis of the scale (p < .01). This can be considered evidence of item discrimination [28,62,63]. To test the reliability of the adolescent individuals’ healthy eating attitude scale, Cronbach’s alpha coefficient, Spearman-Brown (Split-half), and Guttman Lambda-6 coefficients were examined, and these values were above .70. A reliability coefficient above .70 can be considered evidence of the scale’s reliability [26,33,56–58]. The critical value for the total test correlation is specified as .30 [56,64]. In this study, these values were found to be above .30. In addition, when examining the “alpha value when an item is removed,” it was observed that removing any item does not increase the alpha value; therefore, it can be stated that all items contribute positively to the overall internal consistency of the scale and that all items contribute consistently to the overall structure of the scale. It has been noted that the convergent and discriminant validity criteria for the scale should be AVE ≥ .50 and CR ≥ .6 [65]. The values observed in all sub-dimensions were above the criteria, indicating that the discriminant validity is high. Additionally, the square roots of the AVE values were found to be greater than the correlation coefficients. In this case, it can be said that the scale has good discriminant validity. In the analyses of demographic characteristics in the scale data, normality analyses were first performed, and skewness and kurtosis values were examined. In social sciences, skewness and kurtosis values between +1.5 and −1.5 are considered to indicate a normal distribution [42]. Subsequently, an independent t-test, which is one of the parametric tests, was performed for the gender variable. As a result of this test, no statistically significant difference was found in any of the scale’s subdimensions or in the overall mean (p > .05). A review of the literature revealed similar studies conducted in the same culture [66–70], supporting our findings. However, studies with dissimilar results [71] were also found in the literature (p < .05). This difference is thought to stem from sample differences.

## Conclusion

In this study, EFA, CFA, reliability, and validity analyses were performed to develop an attitude scale toward healthy eating for adolescents. The findings reflected the emerging factor structure, thereby strengthening the structural validity. In this context, the attitude scale toward healthy eating developed for adolescents can be said to be reliable, as it provides consistent results across different age groups and grade levels. The scale is both practical (concise and applicable) in terms of application and acceptable to educational institutions. In summary, based on all these data, it can be stated that the attitude scale toward healthy eating for adolescents has been developed as a valid and reliable measurement tool.

### Limitations

This study is limited to individuals aged 11–17, residing in Türkiye and speaking Turkish. It includes only students enrolled in middle and high schools, and its scope is confined to the statistical analyses conducted and the resulting findings.

### Suggestions

Future research could include large-scale cross-cultural studies. Statistical methods could be diversified. The sample structure could be expanded to include different age, gender, socioeconomic status, and regional groups. Cross-validation of the scale could be performed using behavioral measures such as dietary diaries and other data.

## Supporting information

S1 Table (DOCX)
