# Supplementary material for: Adolescents’ attitudes towards healthy eating: A scale development study
Source: PLoS One. 2025 Oct 27;20(10):e0334945. doi: 10.1371/journal.pone.0334945 (PMC12558527; doi:10.1371/journal.pone.0334945)
Supplement: S1 Table — (DOCX) [file pone.0334945.s001.docx]

EK: Ölçeğin Türkçe ana dilinde maddeleri ve faktör yükleri

| Alt boyutlar | Nihai  Madde no  (AFA) | Maddeler | Faktör1 | Faktör 2 | Faktör3 | Faktör 4 | Ortak Faktör Varyansı  (Communalities) |
| --- | --- | --- | --- | --- | --- | --- | --- |
| **Duyuşsal alt boyutu** | M9 | Dengeli ve yeterli beslenme kişiyi olumlu etkiler. | .890 |  |  |  | .655 |
|  | M7 | Dengeli ve yeterli beslenme herkes için önemlidir. | .748 |  |  |  | .558 |
|  | M24 | Dengeli beslenme enerjik hissettirir. | .703 |  |  |  | .618 |
|  | M25 | Besin ögeleri sağlıklı yaşamam için önemlidir. | .677 |  |  |  | .534 |
|  | M30 | Dengeli beslenme bağışıklık sistemimi güçlendirir. | .610 |  |  |  | .521 |
| **Olumlu davranışsal alt boyutu** | M55 | Meyve yemekten hoşlanırım. |  | .787 |  |  | .631 |
|  | M26 | Her öğün meyve tüketince mutlu olurum. |  | .783 |  |  | .510 |
|  | M12 | Haftanın en az 3-4 günü meyve tüketmek benim için önemlidir. |  | .746 |  |  | .600 |
|  | M19 | Dengeli beslenme için meyve tüketmeyi severim. |  | .654 |  |  | .583 |
|  | M56 | Haftada en az 3 gün sebze tüketirim |  | .538 |  |  | .411 |
| **Bilişsel alt boyutu** | M53 | Karbonhidrat içeren gıdaları bilirim. |  |  | .861 |  | .728 |
|  | M52 | Protein içeren gıdaları bilirim. |  |  | .837 |  | .689 |
|  | M54 | Vitamin. mineral içeren gıdaları bilirim. |  |  | .807 |  | .686 |
|  | M23 | Hangi vitaminlerin hangi meyvede olduğunu bilirim. |  |  | .581 |  | .410 |
| **Olumuz davranışsal alt boyutu** | **M34* | *Şekerli besinler tüketmeye bayılırım.* |  |  |  | .839 | .697 |
|  | **M5* | *Abur cubur (çikolata. Cips, büskivi vb.) yiyince mutlu olurum.* |  |  |  | .786 | .632 |
|  | **M49* | *Tatlıyı çok fazla tüketmek hoşuma gider.* |  |  |  | .762 | .599 |
|  | **M17* | *Gazlı içecekleri (kola, enerji içeceği vb.) severim.* |  |  |  | .752 | .568 |
|  |  | Öz Değerler | 5.118 | 2.267 | 1.879 | 1.367 |  |
|  |  | Varyans %’si | 28.43 | 12.59 | 10.43 | 7.59 |  |
|  |  | Toplam Varyans % | 59.05 |  |  |  |  |
|  | ** İşaretli maddeler ters maddelerdir.* | | | | | | |
